# The Effects of Pro-, Pre-, and Synbiotics on Muscle Wasting, a Systematic Review—Gut Permeability as Potential Treatment Target

**DOI:** 10.3390/nu13041115

**Published:** 2021-03-29

**Authors:** Sandra J. van Krimpen, Fleur A. C. Jansen, Veerle L. Ottenheim, Clara Belzer, Miranda van der Ende, Klaske van Norren

**Affiliations:** 1 Nutritional Biology, Division of Human Nutrition, Wageningen University, 6708 WE Wageningen, The Netherlands; sandravk6@hotmail.com (S.J.v.K.); fleur.jansen@wur.nl (F.A.C.J.); veerle.ottenheim2@gmail.com (V.L.O.); miranda.vanderende@wur.nl (M.v.d.E.); 2 Laboratory of Microbiology, Wageningen University, 6708 WE Wageningen, The Netherlands; clara.belzer@wur.nl

**Keywords:** muscle wasting, cachexia, sarcopenia, probiotics, prebiotics, *Lactobacillus*, intestinal permeability, gut–brain axis

## Abstract

Muscle wasting is a frequently observed, inflammation-driven condition in aging and disease, known as sarcopenia and cachexia. Current treatment strategies target the muscle directly and are often not able to reverse the process. Because a reduced gut function is related to systemic inflammation, this might be an indirect target to ameliorate muscle wasting, by administering pro-, pre-, and synbiotics. Therefore, this review aimed to study the potential of pro-, pre-, and synbiotics to treat muscle wasting and to elucidate which metabolites and mechanisms affect the organ crosstalk in cachexia. Overall, the literature shows that *Lactobacillus species pluralis* (spp.) and possibly other genera, such as *Bifidobacterium*, can ameliorate muscle wasting in mouse models. The beneficial effects of *Lactobacillus* spp. supplementation may be attributed to its potential to improve microbiome balance and to its reported capacity to reduce gut permeability. A subsequent literature search revealed that the reduction of a high gut permeability coincided with improved muscle mass or strength, which shows an association between gut permeability and muscle mass. A possible working mechanism is proposed, involving lactate, butyrate, and reduced inflammation in gut–brain–muscle crosstalk. Thus, reducing gut permeability via *Lactobacillus* spp. supplementation could be a potential treatment strategy for muscle wasting.

## 1. Introduction

Muscle wasting is a frequently observed condition that contributes to progressive functional impairment, psychologic distress, and overall reduced resilience [1,2]. Normally, the equilibrium between protein synthesis and breakdown is tightly regulated and influenced by external stimuli such as physical activity and protein intake. However, during muscle wasting, this equilibrium shifts toward muscle protein breakdown, which is often driven by inflammation, either disease- or age-induced. These inflammation-related muscle wasting syndromes are known as cachexia and sarcopenia, respectively [2,3]. Because chronic inflammatory diseases such as cancer primarily develop in the elderly, sarcopenia and cachexia can also co-occur [1,2]. Both syndromes negatively affect life expectancy, survival, and quality of life; however, especially for cachexia, current treatment strategies are limited, palliative, and often not able to reverse the muscle wasting process [1,3].

Current treatment strategies may not be effective yet, as they primarily focus on directly increasing muscle mass. However, not only muscle but also other organs are affected by cachexia such as the brain, kidneys, and gut [1,4]. Communication between these organs is mediated by inflammatory mediators and results in the disturbance of core processes such as appetite regulation, stress, and energy homeostasis [5]. These processes are all closely related to gut function because nutrient absorption, secretion of appetite-regulating hormones, and immune responses are involved. Thus, a disturbed gut function might play a central role in the organ crosstalk that contributes to cachexia [1,4,5]. In addition, it may also be an important factor in sarcopenia, as in both syndromes, gut barrier dysfunction and changed microbiota composition have been observed [4,6].

An intervention that has been described to support gut function is the administering of pro-, pre-, and synbiotics. Probiotics are defined as live microorganisms that confer health benefits when administered in adequate amounts, whereas prebiotics are substrates that are selectively utilized by host microorganisms conferring a health benefit [7,8]. Synbiotics refers to the mixture of pro- and prebiotics that positively affects the beneficial microorganisms in the gut [9]. The mechanisms for their positive effect on gut function still need to be elucidated; however, they may restore gut barrier dysfunction [7]. As more microbiota-related proinflammatory compounds can enter the body when gut barrier function is disrupted, gut permeability could be an important contributor to the inflammatory state during cachexia [5]. Because gut barrier dysfunction has indeed been observed in both cachectic mice and patients [10,11], decreasing gut permeability might be part of the mechanisms via which probiotics could ameliorate muscle wasting. Therefore, the aim of this review was to study the potential of pro-, pre-, and synbiotics to treat muscle wasting and to investigate the association between gut permeability and muscle mass or function.

## 2. Methods

A systematic search was conducted in the databases PubMed and Scopus for all studies published up to 28 October 2020 that used pro-, pre-, and synbiotics to treat muscle wasting and/or function loss in human or animal models of disease or aging. The following advanced search was applied in PubMed (All Fields) and Scopus (TITLE-ABS-KEY): (probiotic* OR prebiotic* OR symbiotic* OR synbiotic*) AND (mice OR male OR female OR men OR women OR patient OR human OR animal) AND (infect* OR tumor OR tumour OR cancer OR disease OR ag*ing) AND (“muscle mass” OR “muscle function” OR “muscle strength” OR “muscle wasting” OR “muscle weakness” OR sarcopen* OR cachexi* OR cachec*). By including the terms “ag*ing” and “sarcopen*,” data on age-induced muscle wasting were also covered and could thus be compared to data on disease-induced muscle wasting. Study selection and data extraction were performed independently by three researchers.

Additionally, a systematic search was conducted for all studies published up to 28 October 2020 that measured gut permeability as well as muscle mass or muscle strength in human or animal models of disease or aging. The following advanced search was applied: (“gut permeability” OR “leaky gut” OR “intestinal permeability” OR “gut homeostasis” OR “intestinal homeostasis” OR “gut barrier”) AND (“skeletal muscle” OR “muscle mass” OR “muscle atrophy” OR “muscle function” OR “muscle strength” OR “muscle weakness” OR cachexi* OR cachec*). This search and its study selection and data extraction were performed similarly to the first systematic search.

## 3. Results

**Figure 1:**
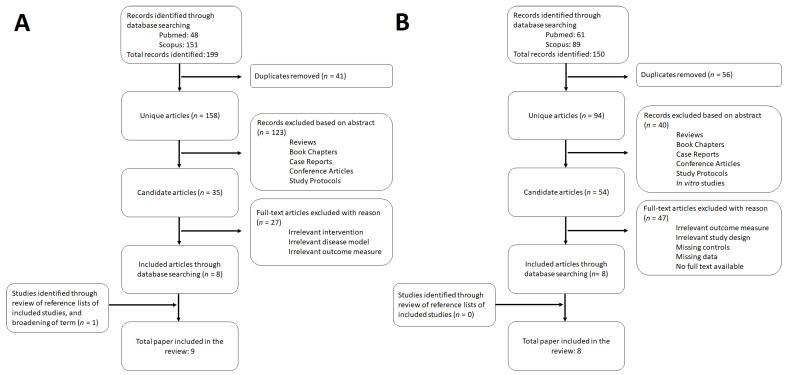
Flow diagram of (**A**) the identified, screened, and included studies on the effect of pro-, pre-, and synbiotics on muscle wasting [12,13,14,15,16,17,18,19,20,21] and (**B**) on the relationship between gut permeability and muscle wasting [15,16,20,22,23,24,25,26].

Over the last five years, the application of pro-, pre-, and synbiotics as disease treatment has substantially gained attention. Due to their effect on gut function, which could influence the multiorgan crosstalk, the use of pro-, pre-, and synbiotics could also be effective to treat cachexia. However, an overview regarding the exact effect of both pro-, pre-, and synbiotics on muscle wasting was lacking. Therefore, a systematic search was conducted to determine whether pro-, pre-, and synbiotics can be used as a treatment for muscle wasting (Figure 1A). This search resulted in 48 articles in PubMed and 151 articles in Scopus, which, together, made a total of 199 articles (Figure 1A). Of those articles, 41 duplicates were removed. After checking their reference lists, we excluded reviews, book chapters, case reports, conference articles, and study protocols, which resulted in 35 research articles being left. These articles were screened fully to assess their eligibility, and eight articles were accepted for inclusion based on exposure, study population, and outcome measures [12,13,14,15,16,17,18,19,20]. After reviewing the reference lists of the included articles and broadening the term “disease” to (“heart failure” OR COPD OR “renal failure” OR HIV), one additional study was included in the review [21]. Moreover, additional research was performed to gain more insights into the relationship between gut permeability and muscle mass or function (Figure 1B). This study selection and data extraction was performed similarly to the first systematic search and resulted in the inclusion of eight studies [15,16,20,22,23,24,25,26].

### 3.1. Effects of Pro-, Pre-, and Synbiotics on Muscle Wasting

All studies selected from the systematic search that included probiotic supplementation were performed in mice and used species from the genus of *Lactobacillus*, except for one that also used a strain of *Bifidobacterium* (Table 1). First of all, Varian et al. [12] found that supplementation with *Lactobacillus reuteri* significantly increased the muscle-to-body weight ratio and muscle fiber size in mice suffering from spontaneous intestinal adenoma. In line with these findings, Bindels et al. [21] showed that supplementation with a combination of *L. reuteri* and *L. gasseri* increased tibialis muscle weight by 8% (*p* = 0.05). However, gastrocnemius muscle weight was not significantly affected. This study was performed in mice injected with BaF cells, mimicking acute leukemia, which is a commonly used model for cancer cachexia. In addition to these studies in cachexia models, four studies with *Lactobacillus species pluralis* (spp.) supplementation in aging models were included. Firstly, Sugimura et al. [13] found that supplementation with *L. lactis* significantly increased the muscle-to-body weight ratio. Secondly, Chen et al. [14] showed that supplementation with *L. paracasei* significantly increased lean body mass. Furthermore, Varian et al. [12] reported that *L. reuteri* supplementation increased both the muscle-to-body weight ratio and muscle fiber size. Lastly, Ni et al. [15] found that *L. casei* significantly increased the muscle-to-body weight ratio as well as forelimb grip strength. Ni et al. [15] was the only study that also assessed the effects of another genus: *Bifidobacterium longum*. These effects were shown to be roughly comparable to the effects of *L. casei*. However, interestingly, the gut microbiota compositions of *Lactobacillus*- and *Bifidobacterium*-treated mice were changed differently upon treatment. For instance, *Lactobacillus* spp. significantly increased upon *L. reuteri* supplementation, but did not change upon B. longum supplementation. In summary, these studies collectively show that supplementation with *Lactobacillus* spp., and possibly also *Bifidobacterium* spp., has the potential to reduce cancer-induced and aging-induced muscle loss.

Furthermore, three studies on prebiotics were obtained from the systematic search, of which one was a randomized controlled clinical trial (Table 1). Firstly, supplementation with pectic oligosaccharides (POS) did not affect muscle mass in a mouse model of neuroblastoma. POS supplementation did also not change *Lactobacillus* spp. abundance in these diseased mice [16]. Secondly, Bindels et al. [17] also studied the effects of POS and, next to that, the effects of inulin in mice injected with BaF cells, inducing acute leukemia. In both groups, no treatment effect on muscle mass or *Lactobacillus* spp. abundance was found. Unfortunately, in the above-mentioned mice studies on prebiotics, cancer development failed to induce loss of muscle mass, which indicated there was no cachexia. Additionally, these prebiotic fibers have been tested in the elderly. A randomized controlled trial focusing on frailty in people over 65 years showed that supplementation with inulin and fructooligosaccharides (FOS) for 13 weeks resulted in reduced exhaustion and increased hand grip strength [18]. This indicates that prebiotics may increase muscle function. Unfortunately, muscle mass was not measured, so it remains unclear whether prebiotics can increase muscle mass in frail elderly.

Lastly, two studies were included focusing on synbiotics (Table 1). The effects of kimchi, a fermented product containing *Leuconostoc mesenteroides* and *Lactobacillus plantarum*, were tested in mice injected with adenocarcinoma [19]. Kimchi was found to significantly increase muscle mass in these mice. Interestingly, reduced expression and serum levels of interleukin (IL)-6 were also found, which is a proinflammatory cytokine involved in cachexia progression. Further, the effects of a combined treatment of *L. reuteri* with oligofructose were studied in mice suffering from acute leukemia [20]. This treatment significantly increased the percentage of lean body mass. So, prebiotics may produce a synergistic effect when combined with probiotics. This section may be divided by subheadings. It should provide a concise and precise description of the experimental results, their interpretation, and the experimental conclusions that can be drawn.

### 3.2. Gut Permeability and Muscle Wasting

As mentioned before, reducing gut permeability is hypothesized to be an effect of probiotic supplementation that may contribute toward the amelioration of muscle wasting. Therefore, a systematic search was performed to investigate the association between gut permeability and muscle mass or function. In total, eight studies were included, measuring both gut permeability and muscle mass or function in disease and aging models (Table 2) [15,16,20,22,23,24,25,26]. All studies were performed in mice, except for the studies of Cuoco et al. [22], Qi et al. [25], and van der Meij et al. [26], which concern human observational studies using matched controls to compare with patients and a young group to compare with elderly. Gut permeability was determined by measuring either the mRNA expression of one or multiple tight junction genes, levels of tight junction proteins, gut permeability markers, or by using an inert sugar permeability assay. Muscle mass was determined as the absolute weight of one or multiple muscles in the mice studies, while the human observational study of Cuoco et al. [22] measured muscle mass by Dual-Energy X-ray Absorptiometry (DEXA). Qi et al. [25] and van der Meij et al. [26] were the only studies that measured hand grip strength rather than muscle mass. In general, high gut permeability is associated with lower muscle mass in the majority of the studies [16,20,22,23,24,25], as only Obermüller et al. [17] showed no difference in muscle mass while gut permeability was increased in the diseased group. Van der Meij et al. [26] did not find an increase in gut permeability in cancer patients compared to matched controls but did find a significant negative correlation between small-intestinal barrier function and hand grip strength. Furthermore, four mice studies included an intervention group aiming to decrease gut permeability [15,16,20,24]. In these studies, decreased gut permeability was associated with decreased muscle mass loss. Thus, the results of the systematic search support the hypothesis of a correlation between gut permeability and muscle mass.

## 4. Discussion

The aim of this review was to study the potential of pro-, pre-, and synbiotics to treat muscle wasting. Our systematic literature analysis showed that *Lactobacillus* spp. and possibly other genera, such as *Bifidobacterium*, can ameliorate muscle wasting in mouse models; however, it has not been studied yet in humans. The effect of prebiotics on muscle wasting could not be established, as the disease models in which this was studied did not develop cachexia. Our second literature search showed that reduced gut permeability often coincides with improved muscle mass or strength in both mouse and human models. Therefore, the described effect of *Lactobacillus* spp. on muscle wasting could be mediated via reduced gut permeability. Unfortunately, the included studies on gut permeability did not elaborate on *Lactobacillus* spp. or the possible working mechanism. Therefore, the following paragraphs will discuss the relation between *Lactobacillus* spp. and gut permeability in the context of the multiorgan nature of cachexia.

### 4.1. Lactobacillus spp., Microbiome Composition, and Gut Permeability

As mentioned earlier, gut permeability appears to be an important factor during muscle wasting. Increased gut permeability in cachectic mice can be linked to microbiome imbalance characterized by an increase in bacteria such as *Klebsiella oxytoca* and *Enterobacteriaceae* spp. [20,27,28]. Interestingly, an increase of these bacteria is associated with a decrease in *Lactobacillus* spp. [20,23,28]. Thus, this suggests that *Lactobacillus* spp. plays an important role in microbiome balance and gut permeability. Yet, in cachexia models, little research has been conducted on the effect of *Lactobacillus* spp. on gut permeability. This effect has, however, been studied in in vitro models [29,30,31] and other diseases/conditions such as inflammation and aging [10,11]. Several in vitro studies showed that *Lactobacillus* spp. positively affects gut permeability, as incubation of CaCo2 cells with different strains of *Lactobacillus* spp. restored the transepithelial resistance after induced intestinal barrier impairment [29,30,31]. In line with these findings, Cui et al. [10] showed that mice injected with lipopolysaccharide (LPS) and supplemented with *L. rhamnosus* had a reduced gut permeability compared to the mice supplemented with the placebo. Moreover, they showed that *L. rhamnosus* affected gut permeability via the regulation of tight junction proteins. These findings were further supported by van Beek et al. [11], as they found that supplementation with *L. plantarum* in aging mice led to a thicker mucus layer compared to control, which is a marker of a balanced microbiome. All three of these studies suggest that supplementation with *Lactobacillus* spp. could improve microbiome balance and decrease gut permeability.

The above-mentioned studies were performed either in in vitro or in mice models. In human studies, there is some indirect evidence supporting the findings mentioned above. The in vitro and mice studies directly showed the effect of *Lactobacillus* spp. on gut permeability, while the human studies only showed gut microbiome composition changes. For example, during chronic kidney disease, systemic inflammation can result in an increase in *Enterobacteriaceae* and *Pseudomonadaceae* genera and a decrease in *Lactobacillus* spp., *Prevotella* spp., and *Bifidobacterium* spp. [32]. In these patients, the changes in the microbiome composition are similar to those found in cachectic mice [20,27,28]. Furthermore, Haran et al. [33] found that with increasing frailty, during aging, there was an increase of *Ruminococcus gnavus* and a decrease of the *Lachnospira* spp. and *Ruminococcus* spp. families. Both bacteria are associated with the maintenance of microbiome balance and production of short-chain fatty acids (SCFAs), specifically butyrate [34,35]. Furthermore, the abundance of other butyrate-producing bacteria decreased, while the elderly with better fitness had a high abundance of *Lactobacillus* spp. [33]. All in all, these human studies show unfavorable changes in the microbiota composition, specifically the reduction of *Lactobacillus* spp., upon inflammation. These findings are in line with the microbiota changes in cachectic mice models.

### 4.2. Lactobacillus spp. and Metabolites

As a result of the transition toward a more imbalanced microbiome during cachexia, the gut permeability can increase. A balanced microbiome breaks down indigestible food components into favorable metabolites that can influence body function. However, when the abundance of certain unfavorable species increases, the metabolites produced by the microbiota can differ from those of a balanced microbiome. Several of these metabolites, such as ammonia and hydrogen sulfide, may increase the risk of high gut permeability and consequently inflammation [36]. The gut microbiome balance can be restored using pro-, pre-, and synbiotics because they stimulate the growth of specific, more favorable groups of bacteria. Consequently, the metabolite profile will be influenced, leading to a more beneficial metabolite profile and lower gut permeability.

Both *Lactobacillus* spp. and *Bifidobacterium* spp. are known to have beneficial effects on the metabolite profile. They belong to the group of lactic acid bacteria and thus produce lactate via fermentative processes. This metabolite is often associated with a balanced microbiome because of its immunomodulating properties via suppression of the LPS/Toll-like receptor 4 signaling pathway [37]. Next to lactate, other metabolites that are considered to have a wide range of beneficial effects are the SCFAs. SCFAs, such as acetate, propionate, and butyrate, are produced by the direct bacterial fermentation of complex carbohydrates. In addition to direct fermentation, the products of one bacterium can be further converted by another bacterium, resulting in the indirect production of metabolites. This mechanism is called cross-feeding and especially contributes to butyrate production. For instance, *Lactobacillus* spp. and *Bifidobacterium* spp. produce lactate, as mentioned before, and acetate, which can both be converted into butyrate by other gut bacteria [38,39]. Butyrate is a frequently studied metabolite in microbiome research because of its “butyrogenic effects.” These effects include providing energy for the colon epithelial cells, maintaining the gut barrier functions, and modulating the immune system in an anti-inflammatory manner [40]. In vitro studies on the relation between butyrate and other SCFAs and gut permeability showed that supplementation with these metabolites normalized the transepithelial resistance after induced intestinal barrier impairment [41,42]. Moreover, these studies showed that SCFAs stimulated the formation of tight junctions. This might be a possible mechanism via which probiotics positively affect gut permeability.

Butyrate production is not only increased by probiotics such as *Lactobacillus* spp. and *Bifidobacterium* spp. but probably also by prebiotics, in particular oligosaccharides and inulin-type fructans (ITFs). Both prebiotics were found to increase *Bifidobacterium* spp., which resulted in increased butyrate production via cross-feeding [18,43]. Research has shown that oligofructose and (pectic) oligosaccharides indeed promote the production of butyrate from lactate and acetate [44,45,46]. In addition, ITFs and (pectic) oligosaccharides can also be consumed by certain butyrate-producing bacteria [47,48]. In conclusion, it is hypothesized that the effect of pro-, pre-, and synbiotics on gut and immune function is partly attributed to increased production of lactate and SCFAs, specifically butyrate, both directly as well as via cross-feeding.

### 4.3. Lactobacillus spp., Inflammation, and Organ Crosstalk

**Figure 2:**
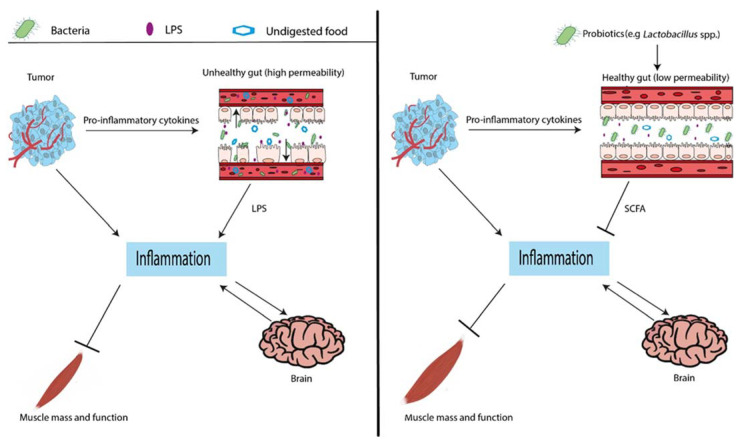
Hypothesis on the mechanism behind the reported effects of probiotics on muscle wasting, involving the organ crosstalk during cancer cachexia. On the left-hand side, the situation when gut permeability is high is illustrated. On the right-hand side, the effect of probiotics is shown. The probiotics inhibit gut permeability and thus improve gut function, reduce inflammation, and consequently ameliorate muscle wasting. LPS: lipopolysaccharide; SCFA: short-chain fatty acid

*Lactobacillus* spp. supplementation was hypothesized to ameliorate muscle wasting by modulating gut permeability and immune function through SCFA production. Because both increased gut permeability and muscle wasting are related to inflammation, this could be a central mediator in the underlying mechanism (Figure 2). IL-6 has especially been described as an important cytokine with respect to these conditions [49]. Several studies on pro- and/or prebiotic supplementation included in Table 1 also measured the levels of IL-6 and other proinflammatory cytokines [13,14,19,21]. In these studies, ameliorated muscle wasting after *Lactobacillus* spp. supplementation was accompanied by decreased levels of these cytokines. This association between inflammation, gut permeability, and muscle wasting is further supported by the observation that injection of anti-IL-6 antibody in cachectic mice leads to a decrease of both gut permeability as well as muscle wasting [23]. So, in general, inflammation and particularly IL-6 can be suggested to play a mediating role in the mechanism via which *Lactobacillus* spp. supplementation ameliorates muscle wasting.

In cachexia, inflammation can stimulate muscle wasting directly, as well as via organ crosstalk. The latter could include the gut–brain axis because this crosstalk is affected by the inflammation resulting from increased gut permeability. Moreover, the gut–brain axis influences core processes involved in muscle wasting such as stress and appetite regulation [5]. Braun et al. [50] found that in cancer cachexia models, hypothalamic inflammation plays a key role in muscle mass loss via the hypothalamus–pituitary–adrenal axis induced production of cortisol. To discover whether influencing these processes could be part of the underlying mechanism, we explored the effects of *Lactobacillus* spp. supplementation on cortisol production and food intake in relation to gut function and inflammation. With respect to cortisol production, Gareau et al. [51] and Ait-Belganoui et al. [52] showed that *Lactobacillus* spp. supplementation decreased corticosterone release as well as gut permeability in stressed rats. In addition, Ait-Belganoui et al. [52] showed in a model with antibiotic-induced disruption of the gut microbiota that increased corticosterone release was a result of more bacterial compounds crossing a disturbed gut barrier. With respect to appetite, decreased levels of IL-6 have been reported to increase food intake. This effect has been associated with the altered expression of inflammation-related genes in the hypothalamus [53]. Contradictorily, supplementation of *Lactobacillus* spp. was found to increase the release of appetite-suppressing hormone glucagon-like peptide (GLP)-1. This effect is thought to be induced via an increased butyrate production [54,55]. When directly assessing the effect of *Lactobacillus* spp. supplementation on food intake in disease and aging, no significant changes were reported (Table 1) [14,15,20,21]. This could be the result of *Lactobacillus* spp. supplementation stimulating appetite via lowering IL-6 on the one hand, while suppressing appetite via stimulating GLP-1 release on the other hand. The effect of *Lactobacillus* spp. stimulating GLP-1 release via butyrate could, however, also potentially improve the delivery and uptake of insulin in the muscle, thereby promoting muscle protein synthesis [56]. Furthermore, GLP-1 agonists have been shown to ameliorate muscle wasting via suppressing myostatin, stimulating myogenic factors, and thus supporting muscle regeneration which has been linked to improved muscle function [57]. Altogether, supplementation of *Lactobacillus* spp. is suggested to ameliorate muscle wasting via increasing butyrate production and decreasing gut permeability, which alters gut–brain interactions and exerts multiorgan effects.

### 4.4. Translatability of Mouse Models

Current findings are foremostly based on mouse studies, and even though the mouse models provide intriguing results, it is important to note that these findings are not directly translatable to humans. Although the gastrointestinal tracts of mice and humans are anatomically comparable, the colon of humans consists of different sections, while in mice it is rather smooth, and there is no division [58]. Moreover, mice have a larger colon than humans when compared to their body weight [59]. These small differences in anatomy can make a big difference in the translatability of the results. Furthermore, many variables can influence the gastrointestinal tract, such as diet, exercise, environment, and stress [59,60]. As these variables are more difficult to control in humans compared to mice, it also makes it harder to translate the results directly. In addition, cancer patients often receive chemotherapy as an anticancer treatment. This has been associated with microbiome imbalance and increased gut permeability, wherefore the chemotherapy may worsen cachexia development. Additionally, it may interfere with interventions that aim to improve microbiome balance such as probiotic supplementation [61]. When the interaction between chemotherapy and probiotic supplementation is further elucidated, an intervention can be developed in which probiotics are combined with other treatment strategies to achieve optimal efficacy. Such multitarget treatment is hypothesized to be more effective because cachexia is a multiorgan syndrome [5].

### 4.5. Future Research

All in all, only a limited number of studies specifically measured the effect of pro-, pre-, and synbiotics on muscle mass or function in cachexia-inducing disease models and could therefore be included in this review. Notably, all included studies on pro- and synbiotics in cachexia used *Lactobacillus* strains. Interestingly, our review showed that although various strains of *Lactobacillus* were used in different cachectic mice models, all studies consistently showed increased muscle mass and function after its supplementation. In addition, the potential of several strains of *Lactobacillus* to reduce muscle wasting was also established in models of age-induced muscle mass loss. This suggests that a generic property of *Lactobacillus* spp. influences muscle wasting regardless of cause. Based on the literature, we hypothesize that the genus of *Lactobacillus*, solely or combined with prebiotics, decreases gut permeability in cachexia by improving gut function via increased lactate and butyrate production. Other lactate-producing bacteria such as *Bifidobacterium* spp. may have similar effects [37], as shown by Ni et al. [15] However, they only investigated age-induced muscle wasting, and the effects of *Bifidobacterium* spp. on disease-induced muscle wasting have not yet been studied. Next to that, it might be of interest to take electrolyte changes into account. In rodent hypertension models, sodium has been reported to influence inflammatory factors both directly as indirectly via the microbiome [62].

To determine whether the effects of pro- and synbiotics are genera- and/or strain-specific and to investigate the underlying mechanism(s), more research is necessary. Future research should focus on the effect of different bacterial genera and strains on microbiome balance, metabolite profiles, gut function, and muscle mass in cachexia and sarcopenia. Based on deviations observed in the microbiota composition of cachectic mice and expected metabolite profile changes [20,27,28], multiple strains of both *Lactobacillus* and *Bifidobacterium* should be investigated in future studies. If these studies show that amelioration of muscle mass loss is mainly driven by improved microbiome balance and gut function rather than by genera-specific effects, other nutritional interventions, such as prebiotics, may also be effective. To improve the validity and comparability of these studies, lean body mass measurements by DEXA are recommended [63]. In the included mice studies, only one or two isolated muscles were measured. Differences occur between studies regarding which muscles are measured and in which units these measurements are expressed. In addition, a few studies define muscle wasting solely based on muscle function, while muscle mass was not taken into account. By measuring total lean body mass by DEXA, validity, as well as comparability, will be increased [64].

## 5. Conclusions

To summarize, *Lactobacillus* spp. has the potential to ameliorate muscle wasting via influencing organ crosstalk, presumably by inducing the production of lactate and butyrate, decreasing the gut permeability, and consequently reducing inflammation. Other genera of bacteria might have similar effects but are not studied yet in relation to muscle mass loss in disease models, although *Bifidobacterium* spp. was found to reduce muscle mass loss in an aging model. To investigate whether the described effects are genus-specific or related to improved gut function in general, more research is needed. Altogether, *Lactobacillus* spp. and possibly other pro-, pre-, and synbiotics have the potential to contribute to effective multitarget cachexia treatment.

## Figures and Tables

**Table 1:** Characteristics and results of the studies included in the systematic research on the effects of pre-, pro- and synbiotics on cachexia.

**Probiotics**							
**Family**	**Source**	**Model**	**Condition**	**Intervention**	**Muscle Outcome**	**Secondary Outcome**	**Reference**
*Lactobacillus*	*reuteri*	C57BL/6 Apc^min/+^ mice	Spontaneous intestinal adenoma	3.5 × 10^5^ CFU/day, 20 weeks	Muscle-to-BW ratio *, fiber size *	Intestinal polyps * and blood neutrophils *	[12]
*Lactobacillus*	*reuteri + gasseri*	BALB/c mice (female)	BaF acute leukemia	2 × 10^8^ CFU/mL drinking water, from disease induction onwards	Muscle (mg)	*Lactobacillus* spp. *, food intake (-), body weight change (-), and IL-6 *	[21]
*Lactobacillus*	*lactis*	SAMP6 mice (female)	Aging (senescence-accelerated)	1 mg/day from 7 to 12 weeks of age	Muscle-to-BW ratio *	Survival *, senescence score *, and IL1beta *	[13]
*Lactobacillus*	*paracasei*	SAMP8 mice (female)	Aging (senescence-accelerated)	1 × 10^9^ CFU/day from 16 to 28 weeks of age	Muscle (% of body) *, muscle strength *	Food intake (-), protein intake (-), TNFalfa *, and IL-6 *	[14]
*Lactobacillus*	*reuteri*	CD-1 mice	Aging	3.5 × 10^5^ CFU/day from 2 to 12 months of age	Muscle-to-BW ratio *, fiber size *	Survival *, blood neutrophils *	[12]
*Lactobacillus*	*casei*	C57BL/6 mice (male)	Aging	2 × 10^9^ CFU/day for 12 weeks from 10 months of age	Muscle-to-BW ratio *, forelimb grip strength *	Food intake (-), fatigue *, gut barrier proteins mRNA *, *Lactobacillus* spp. *, *Bifidobacterium* spp.	[15]
*Bifidobacterium*	*longum*	C57BL/6 mice (male)	Aging	2 × 10^9^ CFU/day for 12 weeks from 10 months of age	Muscle-to-BW ratio *, forelimb grip strength	Food intake (-), fatigue *, gut barrier proteins mRNA *, *Bifidobacterium* spp.	[15]
**Prebiotics**							
	**Type**	**Model**	**Condition**	**Intervention**	**Muscle Outcome**	**Secondary Outcome**	**Reference**
	POS	BALB/c Rj:ATHYM-Foxn1nu/numice (male)	Neuroblastoma	200 mg/day	Muscle/mm (-) (no cachexia developed)	*Lactobacillus* spp. (-), gut permeability (-), food consumption (-)	[16]
	POS	BALB/c mice (male)	BaF acute leukemia	5% POS for 2 weeks	Muscle (mg) (-) (no cachexia developed)	*Lactobacillus* spp. (-), anorexia * and propionate *	[17]
	Inulin	BALB/c mice (male)	BaF acute leukemia	5% inulin for 2 weeks	Muscle (mg) (-) (no cachexia developed)	*Lactobacillus* spp. (-), anorexia *, propionate and butyrate *	[17]
	Inulin + FOS	Elderly (aged 65 and over)	Frailty syndrome	3375 mg inulin + 3488 mg FOS/day for 13 weeks	Hand grip strength *	Energy intake (-), exhaustion *	[18]
**Synbiotics**							
**Probiotic**	**Prebiotic**	**Model**	**Condition**	**Intervention**	**Muscle Outcome**	**Secondary Outcome**	**Reference**
*Leuconostoc mesenteroides* + *Lactobacillus plantarum*	Kimchi	BALB/c mice (male)	C26 colon carcinoma	Normal diet and cpKimchi diet for 3 weeks	Muscle mass *, ubiquitin *, AMPK *, PGC1-a *	Cachexia-induced lipolysis *, lipogenesis *, NF-κB *, AKT *, mTOR *, PI3K * and IL-6 *	[19]
*Lactobacillus reuteri*	OF	BALB/c mice (female)	BaF acute leukemia	2 × 10^8^ CFU/mL probiotic + 0.2 g/day prebiotic from disease induction onwards	Muscle (% BW) *	Energy intake (-), survival (-), and gut barrier proteins mRNA *	[20]

BW: body weight; CFU: colony-forming unit; ↑: increased; (-): no change; ↓: decreased; * *p* < 0.05; POS: polyoligosaccharides; OF: oligofructans.

**Table 2:** Characteristics and results of the studies included in the systematic research on the relationship between gut permeability and muscle mass.

Model	Condition	Type of Intervention	Gut Permeability	Muscle Mass	Reference
BALB/c Rj:ATHYM-Foxn1nu/nu male mice	NB cells	Prebiotics: 200 mg/day oligosaccharides	Gut permeability in NB *, no difference after intervention (-)	Muscle mass in NB (-) (no cachexia developed), no difference after intervention (-)	[16]
Female Balb/c mice	Leukemia (BaF cells)	Synbiotic: inulin-type fructans (0.2 g/day) and *Lactobacillus reuteri* (average: 5.8 × 10^8^ CFU/day)	mRNA expression tight junction genes after BaF injection * mRNA expression tight junction genes after intervention *	Muscle mass after BaF injection *, muscle mass after intervention	[20]
ICR-specific pathogen-free male mice	CKD	FMT	Expression tight junction protein in CKD *, expression tight junction protein after intervention	Muscle mass in CKD *, muscle mass in after intervention *	[24]
Male CD2F1 mice	C26 cells, cancer	N.A.	Gut permeability after C26 injection *	Muscle mass after C26 injection *	[23]
CD1 mice	Aging	Probiotics: *Lactobacillus casei* or *Bifidobacterium longum* (3.5 × 10^5^ CFU/day) from 2 to 12 months of age	mRNA expression tight junction genes in old mice *, mRNA expression tight junction genes after intervention *	Muscle-to-BW ratio in old mice *, muscle-to-BW ratio after intervention * Forelimb grip strength in old mice *, forelimb strength after intervention	[15]
Patients with solid tumors undergoing chemotherapy (*n* = 16)	Cancer	N.A.	Small-intestinal membrane permeability (-)	Muscle strength *	[26]
Newly diagnosed patients (*n* = 13) 17–49 years	Crohn’s disease	N.A.	Gut permeability *	Muscle mass *	[22]
Healthy elderly (*n* = 18) >70 years	Aging	N.A.	Gut permeability *	Muscle strength *	[25]

NB: neuroblastoma; CKD: chronic kidney disease; CFU: colony-forming unit; FMT: fecal microbial transplantation; ↑: increased; (-): no change; ↓: decreased; * *p* < 0.05.

## Data Availability

Not applicable.
